# Fatal amoebic meningoencephalitis caused by *Balamuthia mandrillaris* in *Pongo pygmaeus* and first case report in *Pan troglodytes verus*

**DOI:** 10.3389/fvets.2025.1534378

**Published:** 2025-03-19

**Authors:** Rubén L. Rodríguez-Expósito, Loles Carbonell, Jesús Recuero-Gil, Javier Martinez, Rosa Martinez-Valverde, Carmen Martinez-Fernandez, Joaquín Ortega-Porcel, Agustín Barragán Hernández, Juan M. Corpa, Estefanía Montero Cortijo, Jesús Sánchez-Nicolás, Sergio Moya, Patricia Pérez-Pérez, María Reyes-Batlle, Angélica Domíngez-de-Barros, Omar García-Pérez, Angela Magnet, Fernando Izquierdo, Soledad Fenoy, Carmen del Águila, Elizabeth Córdoba-Lanús, Francisco de Asis García-González, Miguel Casares, José E. Piñero, Jacob Lorenzo-Morales

**Affiliations:** ^1^Instituto Universitario de Enfermedades Tropicales y Salud Pública de Canarias, Universidad de La Laguna, San Cristóbal de La Laguna, Spain; ^2^Departamento de Obstetricia y Ginecología, Pediatría, Medicina Preventiva y Salud Pública, Toxicología, Medicina Legal y Forense y Parasitología, Universidad de La Laguna, La laguna, Spain; ^3^CIBER de Enfermedades Infecciosas (CIBERINFEC), Instituto de Salud Carlos III, Madrid, Spain; ^4^Bioparc Valencia, Valencia, Spain; ^5^Bioparc Fuengirola, Málaga, Spain; ^6^Facultad de Farmacia, Universidad San Pablo-CEU, CEU Universities, Madrid, Spain; ^7^Facultad de Veterinaria, Universidad Cardenal Herrera-CEU, CEU Universities, Departamento de Producción y Sanidad Animal, Salud Pública Veterinaria y Ciencia y Tecnología de los Alimentos, Valencia, Spain; ^8^Myramar Animal Hospital, Fuengirola, Spain; ^9^Hospital Animal Bluecare Partners, Málaga, Spain

**Keywords:** *Balamuthia mandrillaris*, meningoencephalitis, non-human primates, *Pongo pygmaeus*, *Pan troglodytes verus*, multiplex qPCR, IFA assay, Spain

## Abstract

*Balamuthia mandrillaris* is an amoeba that can cause granulomatous amoebic encephalitis (GAE) as well as lung and skin infections in both humans and animals. Studies on *B. mandrillaris*-related GAE cases have increased in recent years. This amoeba has been identified as a cause of encephalitis and death in several non-human primates. In this study, we report a case of a 4-year-old female Bornean orangutan (*Pongo pygmaeus*) in a zoological center that exhibited neurological symptoms for several days. After unsuccessful treatments and a worsening in her condition, euthanasia was deemed necessary. Additionally, we describe the case of a 4-year-old male chimpanzee (*Pan troglodytes verus*) who died suddenly in a different zoo. Postmortem analysis revealed brain lesions with multiple hemorrhages, oedema, and inflammation in various organs in both cases. Histology showed the presence of *B. mandrillaris* trophozoites in necrotic and inflamed brain tissues, consistent with granulomatous amoebic meningoencephalitis. The diagnosis was confirmed using a multiplex qPCR assay on brain tissue samples from both animals water and soil samples from the chimpanzee’s and orangutan’s enclosure tested positive for *B. mandrillaris* DNA by qPCR, confirming environmental exposure. An immunofluorescent antibody (IFA) assay detected *B. mandrillaris* in chimpanzee brain slices. According to the authors’ knowledge, this report documents the first known cases of *Balamuthia* amoebic encephalitis in non-human primates in Spain and the first case in *Pan troglodytes verus*.

## 1 Introduction

Among free-living amoebae (FLA), which are ubiquitous and widely distributed in the environment, only four genera or species are known agents of human and animal infection of the central nervous system (CNS): *Acanthamoeba* spp., *Naegleria fowleri*, *Balamuthia mandrillaris*, and *Sappinia pedata* (1). Amoebic brain infections are an important emerging parasitic disease, mainly in immunodeficient patients. *B. mandrillaris* and several *Acanthamoeba* species can cause granulomatous amoebic encephalitis (GAE), and lung and skin infections (2–5). In contrast, *N. fowleri* causes acute and fulminant primary amoebic meningoencephalitis (PAM), leading in most cases to the death of the patient within a few days (6).

Hitherto, *B. mandrillaris* is an amoeba and the only known species of *Balamuthia* (1). Its life cycle, like *Acanthamoeba*, presents only two stages: a vegetative trophozoite 12–60 μm in size, and a dormant spherical cyst 12–30 μm (1, 3, 7). These protozoans can induce infection directly through contaminated skin lesions, by inhalation via the olfactory nerve, lung and gastrointestinal tract, or organ transplants. This is followed by CNS invasion via haematogenous spread, leading to severe neurological disease. Most patients present with subacute onset of neurological symptoms and rapid progression to death within weeks to years, showing a fatality rate of over 95.0% in most of cases. The high fatality rate may be explained by the delayed diagnosis and ineffective treatment approaches (3, 4, 8).

The first reported case of *Balamuthia mandrillaris* encephalitis occurred in 1986, isolated from a 3-year-old pregnant mandrill monkey (*Mandrillus sphinx*) at the San Diego Wild Animal Park after showing signs of severe paralysis (9). In this regard, the number of studies about *B. mandrillaris* related to GAE cases has gradually increased in recent years. This amoeba has been identified in several non-human primates, such as in western lowland gorilla (*Gorilla gorilla gorilla*) (10–12), Northwest Bornean orangutan (*Pongo pygmaeus pygmaeus*) (13, 14), Kikuyu Colobus monkey (*Colobus guereza kikuyuensis*), or in white-cheeked gibbon (*Nomascus leucogenys*) (12). In addition, cases have been reported in other animals on postmortem examination, including a sheep (15), horses (16), an Indian flying fox (17), a tiger (18) and dogs (19–22).

Herein, we report a case of a 4-year-old female Bornean orangutan whose initial sign was hemiparesis. During the evolution of the symptoms the animal was treated with several different drugs without clinical improvement. A computed tomography (CT) scan revealed multiple masses in the brain. Finally, the orangutan’s condition rapidly progressed to worsening neurological symptoms, and euthanasia was conducted.

On the other hand, we present another case of a 4-year-old male chimpanzee (*Pan troglodytes verus*) without relevant neurological symptoms, who died suddenly in the facilities of the center.

This study summarized the clinical features and detailed the postmortem anatomopathological findings for both cases. The amoebic trophozoites presented in the brain tissue and the multiplex qPCR performed confirmed the final diagnosis of amoebic meningoencephalitis caused by *Balamuthia mandrillaris.* To the authors’ knowledge, this is the first report of *Balamuthia* amoebic encephalitis described in non-human primates in Spain and the first time it has been reported in *Pan troglodytes verus*.

## 2 Case description

### 2.1 Pongo pygmaeus

**Figure 1:**
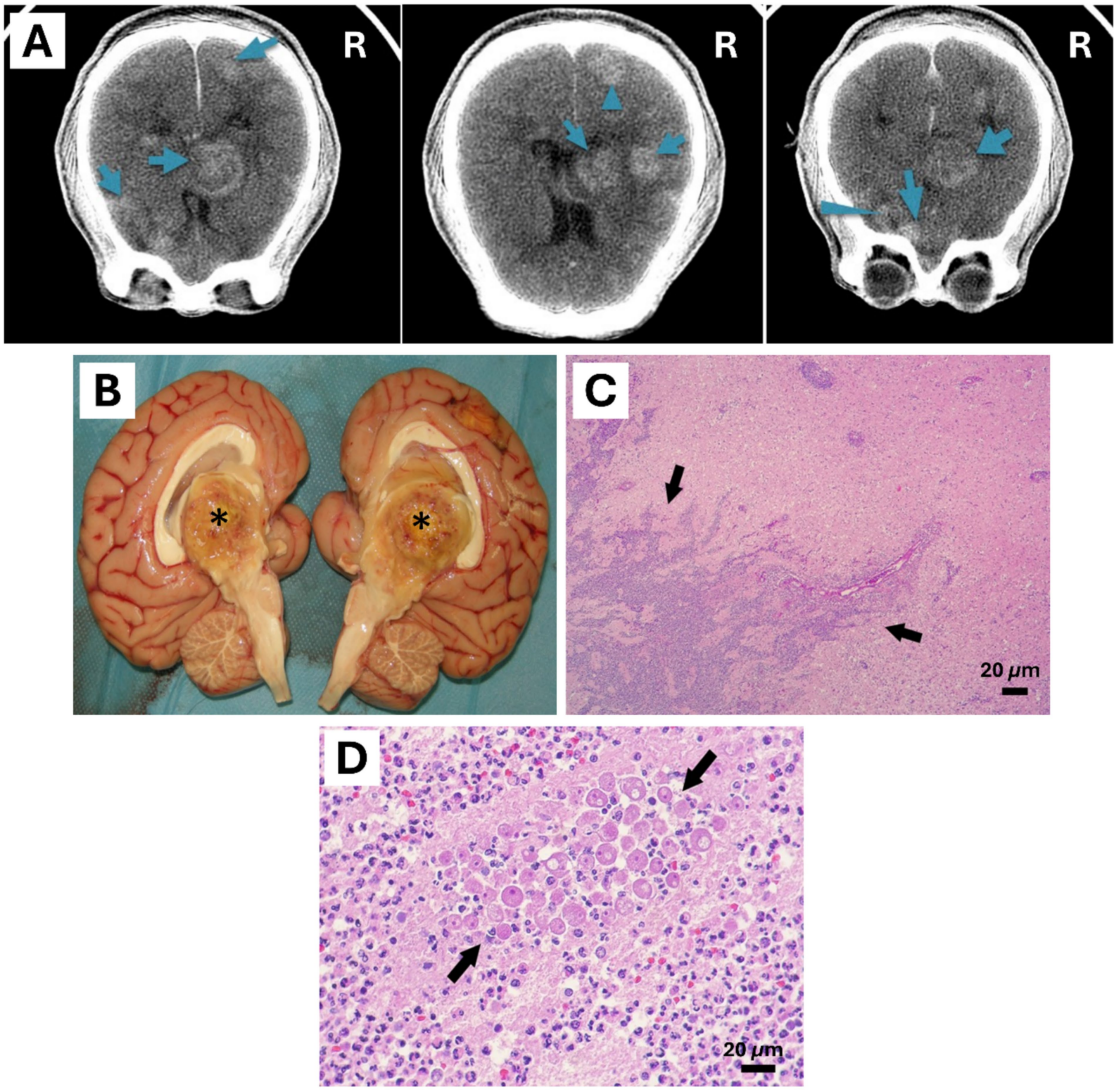
Central nervous system of a 4-year-old female Bornean orangutan (*Pongo pygmaeus*). **(A)** Antemortem CT scan of the brain showed multiple masses mainly located on the right side of the encephalon (masses were marked with blue arrows). **(B)** Mass with central caseous appearance found in the diencephalon and third ventricle, 4 cm in diameter granuloma (*). **(C,D)** Histological findings of the necropsied encephalon. Rounded amoebic trophozoites with granular cytoplasm and prominent 1 to multiple nuclei were observed inside the area of inflammation in the brain (Black arrows). Hematoxylin & Eosin stain (Scale bar represents 20 μm).

In May 2020, a 4-year-old female Bornean orangutan (*Pongo pygmaeus*) from Bioparc Fuengirola developed acute neurological symptoms: hemiparesis of the left arm and leg and subtle paresis of the left side of the mouth. Head injury was initially suspected due to the acute presentation of symptoms, and treatment with dexamethasone was initiated orally to avoid separating the infant from her mother, but without clinical improvement. On the fifth day after the onset of symptoms, the patient was anaesthetized for a computed tomography (CT) scan, which revealed multiple masses in the brain, predominantly on the right side of the brain (Figure 1A). Blood test values were normal, except for a marked increase in alkaline phosphatase. In addition, thoracic and abdominal CT scans, X-rays and ultrasound scans showed no abnormalities. A broad-spectrum serological test for pathogens causing multifocal encephalitis was performed at the Biomedical Primate Research Centre (BPRC, NL), without any positive results for the main agents causing neurological diseases.

Without a definitive diagnosis, treatment was started for the most common agents that can cause multifocal encephalitis: nematodes, cestodes, protozoa, fungi, viruses and bacteria. Antibiotics (cefixime, ceftazidime, rifampicin), antifungals (itraconazole), anti-toxoplasma (pyrimethamine, clindamycin, folinic acid), antiparasitics (praziquantel and albendazole), and antiprotozoals (metronidazole) were administered. Moreover, she was supplemented with corticosteroids from the first day to control inflammation and potential brain oedema. Levetiracetam and diuretics (furosemide and mannitol) were also administered to control status epilepticus, which appeared within a week of the onset of symptoms. However, neurological signs worsened over the days, with seizures, progressive paresis, strabismus, blindness and a comatose state 16 days after the onset of symptoms. Finally, euthanasia was conducted.

Postmortem study was performed at Bioparc fuengirola. Main lesions were found in the brain. Meninges were slightly congestive, multiple yellowish masses with a caseous center, some visible on the brain’s surface. The largest mass of 4 cm in diameter was located in the right side of the third ventricle and diencephalon (Figure 1B). Other smaller ones: 1.5 cm mass in the right frontal lobe, 1.5 cm mass in the right caudal lobe, 1.5 cm mass in the left anterior frontal lobe, 2 cm mass in the left frontal parietal lobe, and a 1.1 cm mass in the right posterior occipital lobe. The remaining organs showed no macroscopic lesions. Likewise, no skin lesions were found. Formalin fixed tissues were sent to Noah’s Path for histopathology study, frozen tissue samples were preserved to allow for further diagnostics.

The macroscopically observed brain lesions corresponded with large areas of necrosis (malacia), infiltrated by vacuolized macrophages (gitter cells), and other areas of intense neutrophilic infiltration. These foci showed peripheral areas of perivascular and neuropil infiltration by numerous lymphocytes, plasma cells and macrophages, and sparse multinucleated giant cells. At the perivascular level, giant cells often formed multiple concentric layers. The suppurative and lymphoplasmocellular-histiocytic inflammation involved the tunica adventitia and even extended to the media of the blood vessels. In addition, fibrin deposits as well as foci of acute hemorrhage could be distinguished in these areas. Numerous blood vessels, particularly in areas of advancing necrosis, contained fibrin thrombi. On the other hand, the cerebellum was unaffected, except for occasional mild perivascular lymphoplasmacytic infiltrates in the meninges.

Regarding to histological findings, gliosis was seen with numerous prominent gemistocytic astrocytes. Round and oval amoebic trophozoites with one or rarely 2 to multiple small, eccentric basophilic or eosinophilic nuclei and abundant cytoplasm containing one or more clear perinuclear vacuoles were distinguished intralesional. These trophozoites presented a size approximately of a 14 to 30 μm in greatest diameter and were concentrated around the adventitia of blood vessels (Figures 1C,D). In larger areas of lesion, particularly in areas of malacia, trophozoites were scarcely visible, while in other areas, especially foci of suppurative inflammation, they were abundant.

Different other lesions, all considered incidental, were also observed in the rest of the organs. In the colon, foci of acute hemorrhage were found scattered in the lamina propria of the mucosa. Furthermore, in the small intestine, moderate hemosiderin deposits were observed. A diffuse pattern was observed in the liver with mild hepatocellular lipidosis. A diffuse pattern was also observed in the adrenal glands, and the cells in the fasciculate area showed high vacuolation in their cytoplasm. In the thyroid gland, the interstice was infiltrated by numerous hypertrophic adipocytes. In the lymph nodes, diffuse depletion of lymphoid tissue was observed, and a dilatation of the medullary sinuses. Finally, numerous cardiomyocytes with mild to moderate hypertrophy of their nuclei were observed in the heart.

### 2.2 Pan troglodytes verus

In November 2022 a 4-year-old male chimpanzee (*Pan troglodytes verus*) was referred already dead to the Clinical Veterinary Hospital of the CEU Cardenal Herrera University by the veterinarians of Bioparc Valencia. On 20 October, the first symptoms appeared, presenting apathy, anorexia and fever. He was administered ibuprofen 7 mg/kg every 12 h for only 3 days, due to the improvement of the symptoms and the fact that his body condition was adequate. On 24 October, he presented a right unilateral strabismus without any other complication. Twelve days after the onset of symptoms, the animal died suddenly in the facilities. No tests or diagnostic procedures were performed during the development of the symptoms, only a postmortem CT scan was performed, but no pathology was observed.

**Figure 2:**
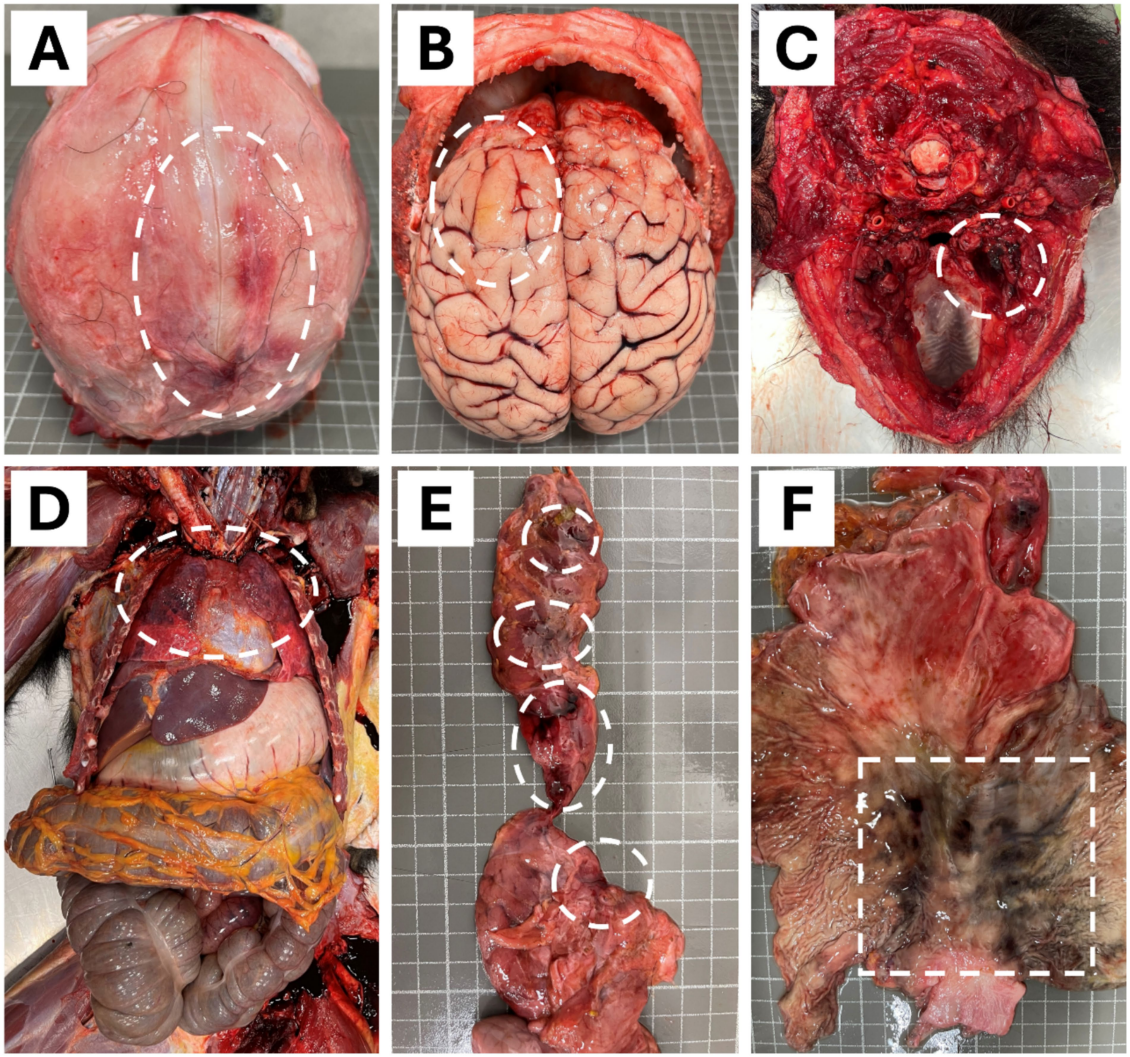
Macroscopic findings in the necropsy of a 4-years-old male chimpanzee (*Pan troglodytes verus*). **(A)** Occipital bone presented hemorrhage lesions of 0.5 to 2 cm on the right side. **(B)** Encephalon showed a delimited congestive and diffuse yellowish areas in the frontal lobe of the left hemisphere. **(C)** Hemorrhage on the inner side of the caudal part of the right mandible. **(D)** Obscured, slightly enlarged, diffuse areas with moderate congestion and oedema in the dorsal portion of both lungs. **(E)** Reddish areas of 0.5 to 1 cm in diameter with multifocal distribution on the pancreas. **(F)** Blackish areas of 1 to 2 cm in diameter with multifocal distribution and poorly defined contours on the gastric mucosa. Circles and a square in the images depicted all lesions.

Postmortem examination revealed that the occipital bone on the right side showed several reddish areas, 0.5–2 cm in diameter, compatible with hemorrhages (Figure 2A). The most severe lesions in this chimpanzee were observed in the brain. The meninges were congestive, and the surface of the brain showed diffuse yellowish areas, slightly elevated in the frontal lobe of the left hemisphere (Figure 2B). In addition, a hemorrhage was observed on the inner side of the caudal part of the right mandible during the necropsy of the animal (Figure 2C).

In the thoracic cavity, both lungs were partially obscured in their dorsal portion, slightly enlarged, diffusely, with moderate congestion and oedema (Figure 2D). Besides this, the pancreas showed reddish areas, 0.5 to 1 cm in diameter, with multifocal distribution (Figure 2E). The gastric mucosa showed blackish areas, 1–2 cm in diameter, with multifocal distribution and poorly defined contours (Figure 2F). The pathology and findings in both organs are compatible with hemorrhages (ecchymosis). The kidneys showed congestion and mild interstitial nephritis; the liver also showed mild congestion.

**Figure 3:**
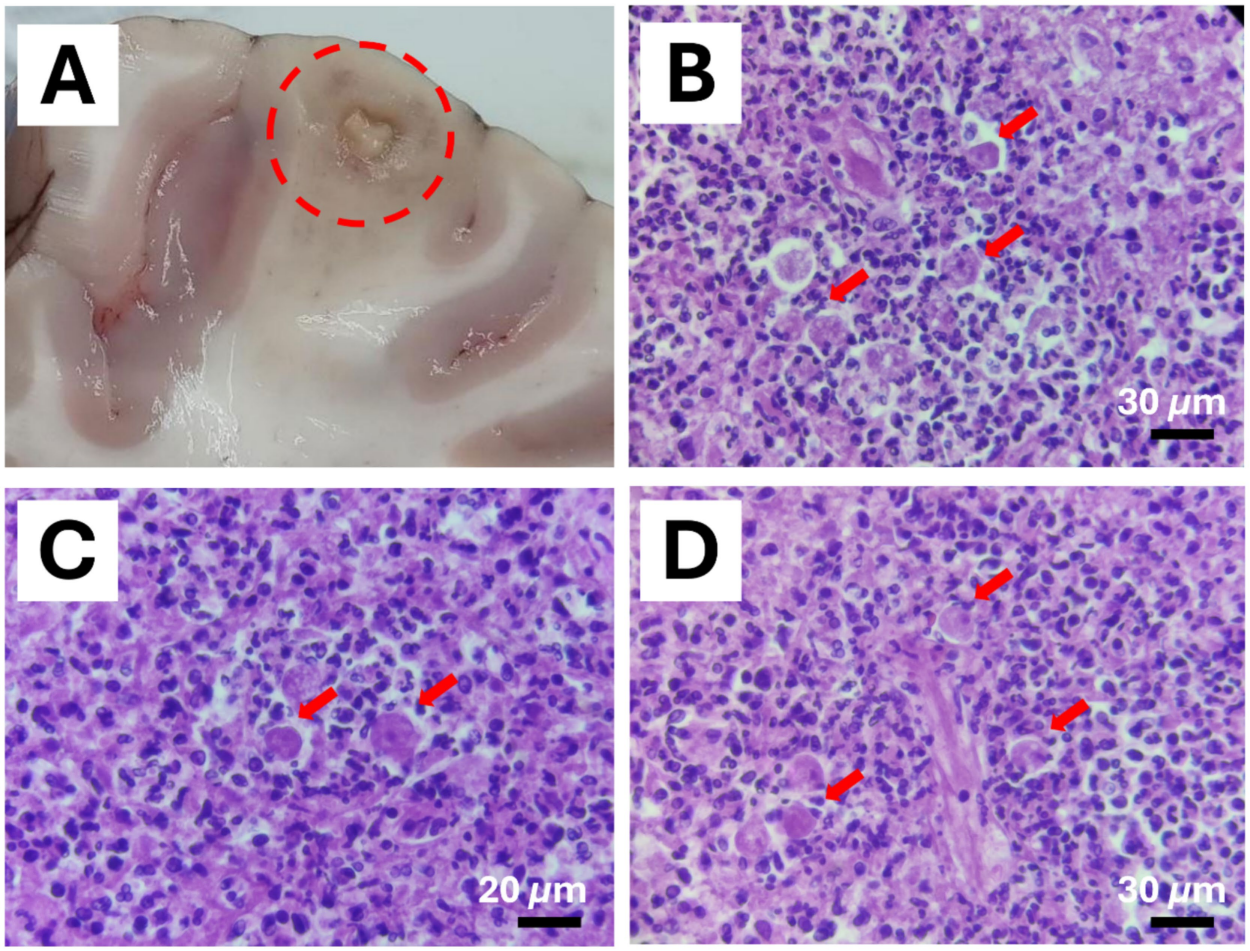
*Balamuthia mandrillaris* brain infection in a 4-years-old male chimpanzee (*Pan troglodytes verus*). **(A)** Granulomatous inflammation in the encephalon (depicted by a circle). **(B–D)** Intralesional polygonal trophozoites with 1 to multiple nuclei and vacuolated cytoplasm were observed (Red arrows). Hematoxylin & Eosin stain (Scale bar represents 30 μm in **B** and **D**; 20 μm in **C**).

Examination of the encephalon revealed multifocal areas of necrosis characterized by yellowish color and granular or cavitated appearance (Figure 3A). Histologically, the brain showed large areas of necrosis with the presence of gitter cells, perivascular cuffs, and inflammation of the meninges. Within the affected areas, parasitic structures were observed with size and morphology consistent with amoebae. Pleomorphic trophozoites with one or multiple nuclei and 12–35 μm in size were observed (Figures 3B–D).

### 2.3 Diagnosis

*Balamuthia mandrillaris* DNA was detected postmortem in brain tissue samples of the orangutan and chimpanzee cases using a multiplex qPCR assay. In addition, the water and soil samples from the facilities where the chimpanzee was housed, and water and soil samples from the orangutan’s facilities were analysed for *B. mandrillaris* DNA detection. The multiplex qPCR assay was performed for simultaneous molecular detection of *Acanthamoeba* spp., *N. fowleri*, and *B. mandrillaris* specific to the region of the 18 s ribosomal RNA gene using the assay conditions and TaqMan probes reported in our previous study (23), with primer sequences first described by Qvarnstrom et al. (24). Cycle threshold (Ct) values of *B. mandrillaris* were 30.4 and 28.7 cycles for orangutan and chimpanzee brain tissue, respectively. Soil and water samples from the chimpanzee facilities were positive to: *Acanthamoeba* spp. (Ct = 31) and *B. mandrillaris* (Ct = 33.9) for the soil, and *Acanthamoeba* spp. (Ct = 31.5) and *B. mandrillaris* (Ct = 33.7) for the water samples. Regarding the soil and water samples from orangutan facilities, multiplex qPCR detected *B. mandrillaris* (Ct = 32.6) in the water samples, and *Acanthamoeba* spp. (Ct = 32.5) and *B. mandrillaris* (Ct = 31.7) in the soil samples.

**Figure 4:**
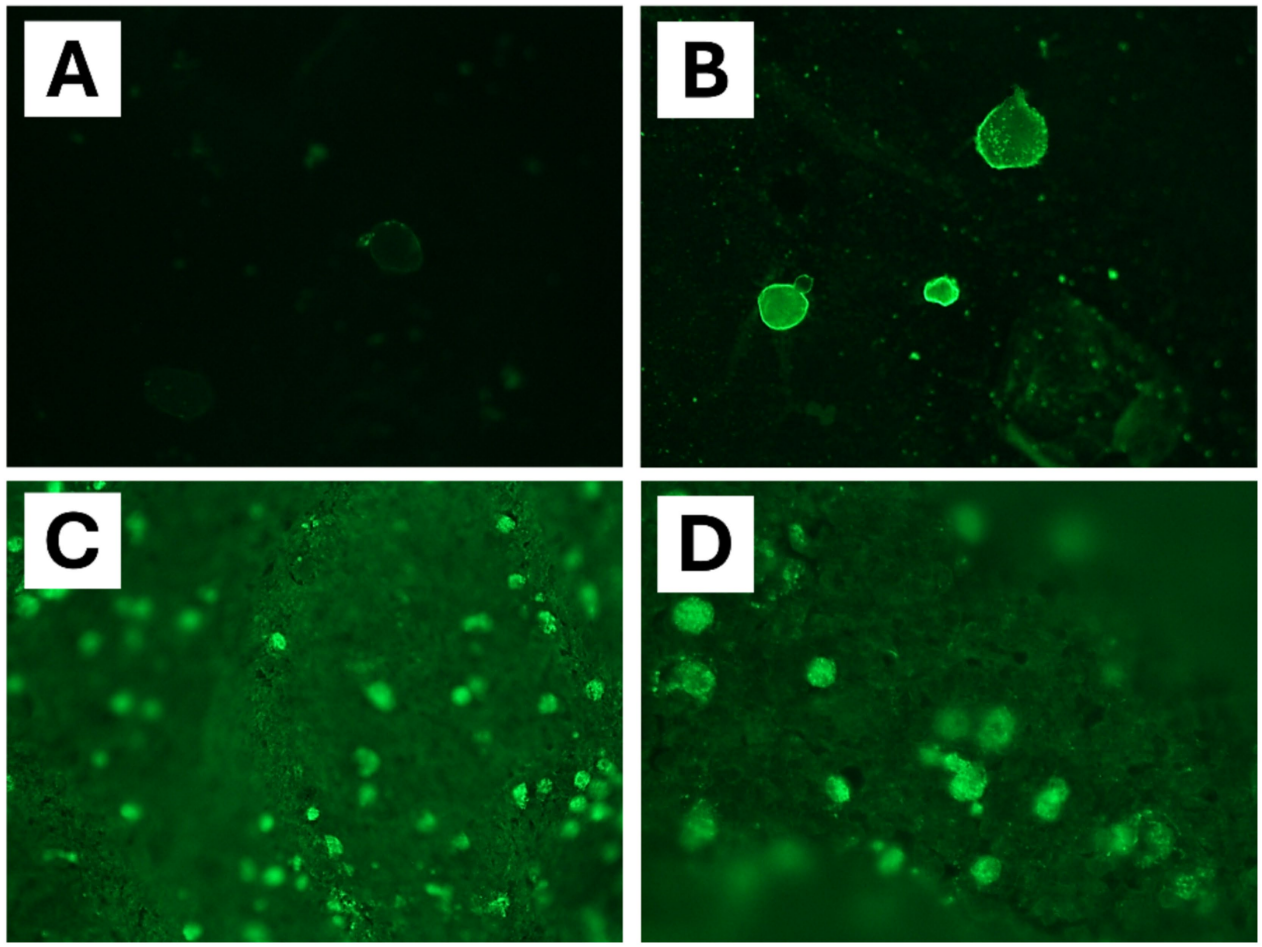
Immunofluorescent antibody (IFA) assay specific for *Balamuthia mandrillaris* detection in brain tissue samples of chimpanzee (*Pan troglodytes verus*). **(A)** Negative control. **(B)** Positive control with cultured amoebae. **(C,D)** IFA assay highlighted the presence of *B. mandrillaris* with bright solid green fluorescence in chimpanzee brain tissues (polyclonal rabbit anti-*Balamuthia* serum).

An immunofluorescent antibody (IFA) assay for detecting *B. mandrillaris* was performed using 5 μm brain slices from chimpanzee. Slides containing the paraffin-embedded tissue section were deparaffinized and incubated with a rabbit polyclonal antibody against *B. mandrillaris.* Amoebae were detected with a fluorochrome-conjugated polyclonal goat anti-rabbit antibody (Sigma F9887; Sigma-Aldrich, Darmstadt, Germany) according to a previously described method (25, 26). IFA assay revealed intense positive green staining of amoebic trophozoites in the chimpanzee brain (Figures 4C,D).

Finally, multiplex qPCR assay combined with clinical features and histological findings supports our final diagnosis of *Balamuthia* amoebic encephalitis in the two cases reported. Furthermore, an IFA assay performed on the chimpanzee brain tissues slides revealed the presence of amoebae, confirming the diagnosis in this case.

## 3 Discussion

Infection produced by *Balamuthia mandrillaris* has been recognized as an emerging disease. Only about 300 cases have been reported worldwide in humans and animals since 1990, mainly in warmer climate regions (3, 7, 27). Most cases have been reported in the US and Peru (27–30), but several cases have also been reported in the Asia-Pacific region (31–35). In this regard, non-human primate cases have been reported in different species. First description of *B. mandrillaris* infection was in a mandrill monkey in the US in 1986 (9). Subsequently, in 1997 a retrospective study revealed this infection in two western lowland gorillas, in a white-cheeked gibbon and in a Kikuyu Colobus monkey in the same zoo as the mandrill monkey (12). In the same year, Canfield et al. (14) reported a case in a Bornean orangutan in New South Wales, Australia. However, it was not until 2011 when Mätz-Rensing et al. (11) reported an infection in Germany in a gorilla. Then, Gjeltema et al. (10) described a new case involving a gorilla in North Carolina in the US. In addition, a second case in an orangutan was reported by Hawkins et al. (13) in the US. In this case report, we describe the first documented case of an infection caused by *B. mandrillaris* in Spain in an orangutan and the first case report describing this infection in a chimpanzee (*Pan troglodytes verus*).

Infection requires environmental exposure to cysts or trophozoites of *B. mandrillaris* and can occur in both immunocompromised and immunocompetent hosts. Certain cases report a history of exposure to soil or water contaminated with this amoeba at animal facilities. In this context, cysts are highly resistant to freezing, temperatures up to 70°C, detergents, ultraviolet radiation, chlorine, and disinfectants (3, 5, 36–39). Therefore, contact with this amoeba through contaminated soil or water in treated or disinfected facilities is still possible. In a previous report, water samples collected in the orangutan facility from *the B. mandrillaris* case infection reported by Hawkins et al. (13) tested real-time PCR positive for this amoeba. In another retrospective study, direct examination of water and soil samples collected in gorilla and mandril enclosures showed several different FLA, but none were identified to species level (12). In the present case, the water and soil samples obtained from the chimpanzee and orangutan facilities revealed the presence of *B. mandrillaris* by multiplex qPCR, suggesting that this could be the source of infection, based on previously reported cases and the biology of amoebae. In this sense, the route of *B. mandrillaris* infection is generally considered to be through skin trauma (3, 40). Nevertheless, the route of exposure in our cases was unknown. No external skin lesions were observed in either the chimpanzee or the orangutan during the examinations or at necropsy. Other reported potential routes of infection include inhalation or even gastrointestinal absorption (41), suggesting these as possible routes of infection in our cases, through the ingestion of water or direct contact with the water source and soil in the facilities.

*B. mandrillaris* infections can have an onset of symptoms that develops over weeks to years as the amoeba replicates in host tissue, eventually causing clinical disease. The infection typically leads to rapid deterioration of the host’s condition, with symptoms such as headache, dysphagia, focal paralysis, seizures, and coma (1–3, 27). In our case, the orangutan primarily exhibited neurological symptoms, including left-sided hemiparesis of the arm and leg, slight facial paresis, seizures, strabismus and confusion that progressed to a comatose state within 2 weeks of symptoms onset. In a previously reported case, an infected orangutan showed symptoms of depression, lethargy, inappetence and head pain (14). More recently, other case reported neurological symptoms, including moderate ataxia, thoracic limb muscle fasciculations, depressed mentation, and reduced fine motor skills, with a progression of years since symptoms onset (13). All these clinical signs align with those observed in our orangutan case, which are typical of *B. mandrillaris* infection. Nonetheless, the chimpanzee case only presented apathy, fever and mild unilateral strabismus, leading to death 12 days after symptoms onset.

Common pathological findings at necropsy include a necrotizing granulomatous encephalitis with infiltration of inflammatory cells and focal hemorrhages. Additionally, histological preparations typically reveal lesions consistent with suppurative necrotizing meningoencephalitis, along with fibrinoid necrosis of small blood vessels, hemorrhage or oedema (3, 4, 10, 13, 14, 28, 42). Both current case reports align with these usual findings. The orangutan and the chimpanzee presented meningoencephalitis with extensive necrotic areas and suppurative inflammation in the affected regions, macroscopically and histologically. Extracranial lesions in hosts infected with *B. mandrillaris* are often rare, but disseminated lesions affecting other organs have been described (2, 19, 20, 28, 30, 31, 41). In our cases, necropsy findings in the chimpanzee included moderate congestion, oedema and hemorrhage in the gastric system, pancreas and lungs. The orangutan showed focal acute hemorrhage in the colon, intestines, liver and adrenal glands, and several infiltrative inflammation and lesions in other regions. However, according to the pathologist, these were mild findings and unrelated to the cause of death. In this context, no secondary cause for these findings was identified in either case. Previously reported cases in orangutans have not documented extracranial lesions associated with this amoebic infection (13, 14).

In most cases, the diagnosis of *B. mandrillaris* infection in non-human primates is usually reported at postmortem. Furthermore, since postmortem analysis of these animals is uncommon in many countries, it suggests that the number of deaths due to *B. mandrillaris* could be significantly higher than reported. A further complication is that diagnosing this amoebic infection usually requires a brain tissue biopsy for confirmatory analysis. *Balamuthia* is rarely detectable in the cerebrospinal fluid of infected hosts, either through microscopy or PCR (4, 8, 13, 18, 26, 43). In our report, a postmortem multiplex qPCR assay was performed on brain tissue samples to confirm the final diagnosis of *B. mandrillaris* infection in both orangutan and chimpanzee cases. Recent advances in molecular biology allow for highly sensitivity and specificity detection of this amoeba in water and soils samples (23, 24). Likewise, we conducted an IFA assay, which revealed stained trophozoites in the chimpanzee brain tissue. These rapid and highly sensitive/specific assays could facilitate earlier antemortem treatment. Concerning this, Ferris et al. (25) studied the presence of *B. mandrillaris* antibodies in stored serum samples from five orangutans using a human IFA assay, suggesting that this may enable novel antemortem diagnosis in individuals with symptoms consistent with an amoebic encephalitis, which is critical for timely treatment.

Optimal treatment for *B. mandrillaris* encephalitis remains a challenge. Delay in diagnosis, misdiagnosis, lack of effective drugs, and the severity of infection, all contributing to the low survival rate (4, 8, 27, 39, 43, 44). Current treatment strategies involve combination therapy with miltefosine, fluconazole, miconazole, ketoconazole, 5-fluorocytosine, macrolides or pentamidine. The Centers for Disease Control and Prevention (CDC) recommends a regimen including pentamidine, sulfadiazine, flucytosine, albendazole, fluconazole, azithromycin and miltefosine based on cases of surviving patients (28, 30, 45). However, there is no specific protocol for treating this parasitic disease. In the present case, the chimpanzee died suddenly, and no therapy was administered. The orangutan, in absence of diagnosis, was treated with combination therapy targeting the most common causes of encephalitis in non-human primates, but this was unsuccessful, leading to euthanasia decision. In this regard, fluoroquinolone nitroxoline has recently been used to successfully treat *B. mandrillaris* encephalitis in a human case (46, 47). Additionally, in previously studies we demonstrate that nitroxoline could inhibit other amoebas that cause encephalitis in humans and animals, *Acanthamoeba* spp. and *Naegleria fowleri* specifically, inducing a programmed cell death in these parasites (48, 49). Therefore, nitroxoline may represent a promising new therapeutic option for developing more effective therapies against *B. mandrillaris.*

## Data Availability

The raw data supporting the conclusions of this article will be made available by the authors, without undue reservation.

## References

[1] Visvesvara GS Moura H Schuster FL. Pathogenic and opportunistic free-living amoebae: *Acanthamoeba* spp., *Balamuthia mandrillaris*, *Naegleria fowleri*, and *Sappinia diploidea*. FEMS Immunol Med Microbiol. (2007) 50:1–26. doi: 10.1111/j.1574-695X.2007.00232.x, PMID: 17428307

[2] Lee DC Fiester SE Madeline LA Fulcher JW Ward ME Schammel CM-G. *Acanthamoeba* spp. and *Balamuthia mandrillaris* leading to fatal granulomatous amebic encephalitis. Forensic Sci Med Pathol. (2020) 16:171–6. doi: 10.1007/s12024-019-00202-6, PMID: 31773473

[3] Bhosale NK Parija SC. *Balamuthia mandrillaris*: an opportunistic, free-living ameba–an updated review. Trop Parasitol. (2021) 11:78–88. doi: 10.4103/tp.tp_36_21, PMID: 34765527 PMC8579774

[4] Berger JR. Amebic infections of the central nervous system. J Neurovirol. (2022) 28:467–72. doi: 10.1007/s13365-022-01096-x, PMID: 36098909

[5] Magnet A Fenoy S Galván AL Izquierdo F Rueda C Fernandez Vadillo C. A year long study of the presence of free living amoeba in Spain. Water Res. (2013) 47:6966–72. doi: 10.1016/j.watres.2013.09.065, PMID: 24200005

[6] Piñero JE Chávez-Munguía B Omaña-Molina M Lorenzo-Morales J. Naegleria fowleri. Trends Parasitol. (2019) 35:848–9. doi: 10.1016/j.pt.2019.06.011 31300281

[7] Zhang Z Liang J Wei R Feng X Wang L Wang L. Facial *Balamuthia mandrillaris* infection with neurological involvement in an immunocompetent child. Lancet Infect Dis. (2022) 22:e93–e100. doi: 10.1016/S1473-3099(21)00334-0, PMID: 34838200

[8] Haston JC Cope JR. Amebic encephalitis and meningoencephalitis: an update on epidemiology, diagnostic methods, and treatment. Curr Opin Infect Dis. (2023) 36:186–91. doi: 10.1097/QCO.0000000000000923, PMID: 37093056 PMC10798061

[9] Visvesvara GS Martinez AJ Schuster FL Leitch GJ Wallace SV Sawyer TK. Leptomyxid ameba, a new agent of amebic meningoencephalitis in humans and animals. J Clin Microbiol. (1990) 28:2750–6. doi: 10.1128/jcm.28.12.2750-2756.1990, PMID: 2280005 PMC268267

[10] Gjeltema JL Troan B Muehlenbachs A Liu L Da Silva AJ Qvarnstrom Y. Amoebic meningoencephalitis and disseminated infection caused by *Balamuthia mandrillaris* in a Western lowland gorilla (*Gorilla gorilla gorilla*). J Am Vet Med Assoc. (2016) 248:315–21. doi: 10.2460/javma.248.3.315, PMID: 26799111

[11] Mätz-Rensing K Kunze M Zöller M Roos C Kiderlen AF Ludwig C. Fatal *Balamuthia mandrillaris* infection in a gorilla–first case of balamuthiasis in Germany. J Med Primatol. (2011) 40:437–40. doi: 10.1111/j.1600-0684.2011.00479.x, PMID: 21496054

[12] Rideout BA Gardiner CH Stalis IH Zuba JR Hadfield T Visvesvara GS. Fatal infections with *Balamuthia mandrillaris* (a free-living amoeba) in gorillas and other old world primates. Vet Pathol. (1997) 34:15–22. doi: 10.1177/030098589703400103, PMID: 9150541

[13] Hawkins SJ Struthers JD Phair K Ali IKM Roy S Mull B. Diagnostic evaluation of fatal *Balamuthia mandrillaris* meningoencephalitis in a captive Bornean orangutan (*Pongo pygmaeus*) with identification of potential environmental source and evidence of chronic exposure. Primates. (2021) 62:51–61. doi: 10.1007/s10329-020-00860-z, PMID: 32920664

[14] Canfield PJ Vogelnest L Cunningham ML Visvesvara GS. Amoebic meningoencephalitis caused by *Balamuthia mandrillaris* in an orangutan. Aust Vet J. (1997) 75:97–100. doi: 10.1111/j.1751-0813.1997.tb14165.x, PMID: 9066963

[15] Fuentealba IC Wikse SE Read WK Edwards JF Visvesvara GS. Amebic meningoencephalitis in a sheep. J Am Vet Med Assoc. (1992) 200:363–5. doi: 10.2460/javma.1992.200.03.363, PMID: 1548173

[16] Kinde H Read DH Daft BM Manzer M Nordhausen RW Kelly DJ. Infections caused by pathogenic free-living amebas (*Balamuthia Mandrillaris* and *Acanthamoeba* sp.) in horses. J Vet Diagn Invest. (2007) 19:317–22. doi: 10.1177/104063870701900318, PMID: 17459867

[17] Crossland NA Ali I Higbie C Jackson J Pirie G Bauer R. Neurologic amebiasis caused by *Balamuthia mandrillaris* in an Indian flying fox (*Pteropus giganteus*). J Vet Diagn Invest. (2016) 28:54–8. doi: 10.1177/1040638715614346, PMID: 26762405

[18] Niedringhaus KD Gordon M Yabsley MJ Gai J Uzal FA Woolard KD. Fatal balamuthosis in a Siberian tiger and a literature review of detection options for free-living amoebic infections in animals. J Vet Diagn Invest. (2023) 35:311–6. doi: 10.1177/10406387231160771, PMID: 36908206 PMC10185987

[19] Foreman O Sykes J Ball L Yang N De Cock H. Disseminated infection with *Balamuthia mandrillaris* in a dog. Vet Pathol. (2004) 41:506–10. doi: 10.1354/vp.41-5-506, PMID: 15347823

[20] Finnin PJ Visvesvara GS Campbell BE Fry DR Gasser RB. Multifocal *Balamuthia mandrillaris* infection in a dog in Australia. Parasitol Res. (2007) 100:423–6. doi: 10.1007/s00436-006-0302-0, PMID: 17033842

[21] Chien RC-C Telford CR Roy S Ali IKM Shieh W-J Confer AW. Canine amoebic meningoencephalitis due to *Balamuthia mandrillaris*. Vet Parasitol Reg Stud Rep. (2018) 13:156–9. doi: 10.1016/j.vprsr.2018.06.003, PMID: 31014865

[22] Hodge PJ Kelers K Gasser RB Visvesvara GS Martig S Long SN. Another case of canine amoebic meningoencephalitis—the challenges of reaching a rapid diagnosis. Parasitol Res. (2011) 108:1069–73. doi: 10.1007/s00436-010-2197-z, PMID: 21161275

[23] Córdoba-Lanús E Reyes-Batlle M Domínguez-de-Barros A Pérez-Pérez P Rodríguez-Expósito RL García-Ramos A. Multiplex real-time polymerase chain reaction assay to detect *Acanthamoeba* spp., *Vermamoeba vermiformis*, *Naegleria fowleri*, and *Balamuthia mandrillaris* in different water sources. Am J Trop Med Hyg. (2024) 111:785–90. doi: 10.4269/ajtmh.24-0028, PMID: 39106847 PMC11448538

[24] Qvarnstrom Y Visvesvara GS Sriram R da Silva AJ. Multiplex real-time PCR assay for simultaneous detection of *Acanthamoeba* spp., *Balamuthia mandrillaris*, and *Naegleria fowleri*. J Clin Microbiol. (2006) 44:3589–95. doi: 10.1128/JCM.00875-06, PMID: 17021087 PMC1594764

[25] Ferris RL Ali IK West G. Use of a human indirect immunofluorescence antibody assay for *Balamuthia mandrillaris* in a group of captive Northwest Bornean orangutans (*Pongo pygmaeus pygmaeus*). J Zoo Wildl Med. (2021) 52:310–4. doi: 10.1638/2019-0018, PMID: 33827191

[26] Schuster FL Honarmand S Visvesvara GS Glaser CA. Detection of antibodies against free-living amoebae *Balamuthia mandrillaris* and *Acanthamoeba* species in a population of patients with encephalitis. Clin Infect Dis. (2006) 42:1260–5. doi: 10.1086/503037, PMID: 16586385

[27] Bravo FG Seas C. *Balamuthia Mandrillaris* amoebic encephalitis: an emerging parasitic infection. Curr Infect Dis Rep. (2012) 14:391–6. doi: 10.1007/s11908-012-0266-4, PMID: 22729402

[28] Cope JR Landa J Nethercut H Collier SA Glaser C Moser M. The epidemiology and clinical features of *Balamuthia mandrillaris* disease in the United States, 1974–2016. Clin Infect Dis. (2019) 68:1815–22. doi: 10.1093/cid/ciy813, PMID: 30239654 PMC7453664

[29] Cabello-Vílchez AM Rodríguez-Zaragoza S Piñero J Valladares B Lorenzo-Morales J. *Balamuthia mandrillaris* in South America: an emerging potential hidden pathogen in Perú. Exp Parasitol. (2014) 145:S10–9. doi: 10.1016/j.exppara.2014.05.007, PMID: 24858923

[30] Centers for Disease Control and Prevention (CDC). Balamuthia amebic encephalitis--California, 1999-2007. MMWR Morb Mortal Wkly Rep. (2008) 57:768–71. 18636064

[31] Wang L Cheng W Li B Jian Z Qi X Sun D. *Balamuthia mandrillaris* infection in China: a retrospective report of 28 cases. Emerg Microbes Infect. (2020) 9:2348–57. doi: 10.1080/22221751.2020.1835447, PMID: 33048025 PMC7599003

[32] Xu H Wang D Cui K Wan R Chi Q Wu T. 18F-FDG PET/CT findings in fatal *Balamuthia mandrillaris* encephalitis in brain stem: a case report. Radiol Case Rep. (2024) 19:1851–4. doi: 10.1016/j.radcr.2024.02.021, PMID: 38425772 PMC10901688

[33] Yamanouchi K Arima H Sakamoto Y Kanto K Kasai K Ito K. First report of the isolation of *Balamuthia mandrillaris* in the northern region of Japan. Parasitol Res. (2018) 117:2895–900. doi: 10.1007/s00436-018-5980-x, PMID: 29961176 PMC6105249

[34] Lee JY Yu IK Kim SM Kim JH Kim HY. Fulminant disseminating fatal granulomatous amebic encephalitis: the first case report in an immunocompetent patient in South Korea. Yonsei Med J. (2021) 62:563–7. doi: 10.3349/ymj.2021.62.6.563, PMID: 34027644 PMC8149926

[35] Intalapaporn P Suankratay C Shuangshoti S Phantumchinda K Keelawat S Wilde H. *Balamuthia mandrillaris* meningoencephalitis: the first case in Southeast Asia. Am J Trop Med Hyg. (2004) 70:666–9. doi: 10.4269/ajtmh.2004.70.666, PMID: 15211011

[36] Cabello-Vílchez AM Reyes-Batlle M Montalbán-Sandoval E Martín-Navarro CM López-Arencibia A Elias-Letts R. The isolation of *Balamuthia mandrillaris* from environmental sources from Peru. Parasitol Res. (2014) 113:2509–13. doi: 10.1007/s00436-014-3900-2, PMID: 24781021

[37] Yamanouchi K Arima H Sakamoto Y Kanto K Itoh K Tsujiguchi T. Isolation and habitat analysis of *Balamuthia mandrillaris* from soil. Parasitol Res. (2024) 123:163. doi: 10.1007/s00436-024-08182-5, PMID: 38499865

[38] Schuster FL Dunnebacke TH Booton GC Yagi S Kohlmeier CK Glaser C. Environmental isolation of *Balamuthia mandrillaris* associated with a case of amebic encephalitis. J Clin Microbiol. (2003) 41:3175–80. doi: 10.1128/JCM.41.7.3175-3180.2003, PMID: 12843060 PMC165348

[39] Siddiqui R Ortega-Rivas A Khan NA. *Balamuthia mandrillaris* resistance to hostile conditions. J Med Microbiol. (2008) 57:428–31. doi: 10.1099/jmm.0.47694-0, PMID: 18349360

[40] Alvarez P Torres-Cabala C Gotuzzo E Bravo F. Cutaneous balamuthiasis: a clinicopathological study. JAAD Int. (2022) 6:51–8. doi: 10.1016/j.jdin.2021.11.005, PMID: 35059659 PMC8760460

[41] Kiderlen AF Laube U Radam E Tata PS. Oral infection of immunocompetent and immunodeficient mice with *Balamuthia mandrillaris* amebae. Parasitol Res. (2007) 100:775–82. doi: 10.1007/s00436-006-0334-5, PMID: 17111178

[42] Hara T Yagita K Sugita Y. Pathogenic free-living amoebic encephalitis in Japan. Neuropathology. (2019) 39:251–8. doi: 10.1111/neup.12582, PMID: 31243796

[43] Parija SC Dinoop K Venugopal H. Management of granulomatous amebic encephalitis: laboratory diagnosis and treatment. Trop Parasitol. (2015) 5:23–8. doi: 10.4103/2229-5070.149889, PMID: 25709949 PMC4326989

[44] Spottiswoode N Haston JC Hanners NW Gruenberg K Kim A DeRisi JL. Challenges and advances in the medical treatment of granulomatous amebic encephalitis. Ther Adv Infect Dis. (2024) 11:20499361241228340. doi: 10.1177/20499361241228340, PMID: 38312848 PMC10838035

[45] Centers for Disease Control and Prevention (CDC) (2024) Clinical care of balamuthia infection. In: Treatment recommendations. Available online at: https://www.cdc.gov/balamuthia/hcp/clinical-care/index.html (accessed November 13, 2024).

[46] Spottiswoode N Pet D Kim A Gruenberg K Shah M Ramachandran A. Successful treatment of *Balamuthia mandrillaris* granulomatous amebic encephalitis with nitroxoline. Emerg Infect Dis. (2023) 29:197–201. doi: 10.3201/eid2901.221531, PMID: 36573629 PMC9796214

[47] Laurie MT White CV Retallack H Wu W Moser MS Sakanari JA. Functional assessment of 2,177 U.S. and international drugs identifies the quinoline nitroxoline as a potent amoebicidal agent against the pathogen *Balamuthia mandrillaris*. mBio. (2018) 9:e02051-18. doi: 10.1128/mBio.02051-18, PMID: 30377287 PMC6212833

[48] Rodríguez-Expósito RL Sifaoui I Reyes-Batlle M Fuchs F Scheid PL Piñero JE. Induction of programmed cell death in *Acanthamoeba culbertsoni* by the repurposed compound nitroxoline. Antioxidants. (2023) 12:2081. doi: 10.3390/antiox12122081, PMID: 38136200 PMC10740438

[49] Chao-Pellicer J Arberas-Jiménez I Fuchs F Sifaoui I Piñero JE Lorenzo-Morales J. Repurposing of nitroxoline as an alternative primary amoebic meningoencephalitis treatment. Antibiotics. (2023) 12:1280. doi: 10.3390/antibiotics12081280, PMID: 37627700 PMC10451279

